# The role of PKAc1 in gene regulation and trichodimerol production in *Trichoderma reesei*

**DOI:** 10.1186/s40694-019-0075-8

**Published:** 2019-09-10

**Authors:** Wolfgang Hinterdobler, André Schuster, Doris Tisch, Ezgi Özkan, Hoda Bazafkan, Johann Schinnerl, Lothar Brecker, Stefan Böhmdorfer, Monika Schmoll

**Affiliations:** 1 Center for Health and Bioresources, AIT Austrian Institute of Technology, Konrad Lorenz Strasse 24, 3430 Tulln, Austria; 2 Institute of Chemical Engineering, Vienna University of Technology, Getreidemarkt 9, 1060 Vienna, Austria; 3 Department of Chemistry, University of Natural Resources and Life Sciences (BOKU), Konrad-Lorenz-Straße 24, 3430 Tulln, Austria; 4 Chemodiversity Research Group, Department of Botany and Biodiversity Research, University of Vienna, Rennweg 14, 1030 Vienna, Austria; 5 Department of Organic Chemistry, University of Vienna, Währinger Strasse 38, 1090 Vienna, Austria

**Keywords:** *Trichoderma reesei*, *Hypocrea jecorina*, cAMP pathway, Cellulase, Trichodimerol, Secondary metabolism, Sexual development

## Abstract

**Background:**

*Trichoderma reesei* represents a model system for investigation of plant cell wall degradation and its connection to light response. The cyclic adenosine monophosphate pathway (cAMP pathway) plays an important role in both physiological outputs, being crucial for regulation of photoreceptor function as well as for cellulase regulation on different carbon sources. Phosphorylation of photoreceptors and of the carbon catabolite repressor CRE1 was shown in ascomycetes, indicating a relevance of protein kinase A in regulation of the target genes of these transcription factors as well as an impact on regulation of induction specific genes. Moreover, the cAMP pathway impacts growth and development.

**Results:**

Here, we investigated gene regulation by the catalytic subunit of protein kinase A (PKAc1) upon growth on cellulose. We found distinct gene sets for regulation upon growth in light and darkness with an overlap of only 13 genes. PKAc1 regulates metabolic genes as well as transport and defense functions. The overlap of gene regulation by PKAc1 with the genes representing the cAMP dependent regulatory output of the photoreceptor ENV1 indicates an involvement of PKA in this pathway, which counteracts its effects by contrasting regulation. Moreover, we found considerable overlap with the gene sets regulated under cellulase inducing conditions and by the carbon catabolite repressor CRE1. Our analysis also showed that PKAc1 regulates the genes of the SOR cluster associated with the biosynthesis of sorbicillinoids. The homologue of *gin4*, encoding a CAMK type kinase, which is regulated by PKAc1, CRE1 and YPR2 showed a moderate impact on trichodimerol production. We isolated trichodimerol as representative sorbicillin compound and established a method for its quantification in large sample sets using high performance thin layer chromatography (HPTLC), which can be broadly applied for secondary metabolite screening of mutants or different growth conditions. Due to the high expression levels of the SOR cluster under conditions of sexual development we crosschecked the relevance of PKAc1 under these conditions. We could show that PKAc1 impacts biosynthesis of trichodimerol in axenic growth and upon mating.

**Conclusions:**

We conclude that PKAc1 is involved in light dependent regulation of plant cell wall degradation, including carbon catabolite repression as well as secondary metabolism and development in *T. reesei*.

## Background

*Trichoderma reesei* is one of the most prolific enzyme producers in industry worldwide and plays a crucial role in expression of plant cell wall degrading enzymes for second generation biofuel production [1, 2]. Therefore, its enzyme system is studied in detail as are the complex regulation systems supporting high efficiency production of homologous and heterologous proteins [3]. Regulation of plant cell wall degrading enzymes is induced in response to carbon sources such as cellulose, lactose and sophorose, the natural inducer, and genes encoding the major enzymes for this purpose are coregulated [3–6]. Numerous transcription factors are involved in adjustment of transcript levels of plant cell wall degrading enzymes to environmental conditions, which can act positively or negatively [7]. In *T. reesei* the most important transcription factors are XYR1 [8] and ACE3 [9], which are essential for cellulase gene expression, ACE1 [10], which negatively affects cellulases and CRE1 [11], the carbon catabolite repressor, which represses cellulase formation in the presence of easily metabolizable carbon sources. Besides regulation at the transcriptional level, recent analyses revealed that cellulase expression is also regulated at a posttranscriptional level in dependence of nutrient sensing via the heterotrimeric G-protein pathway [12].

In addition to the carbon source, also other environmental cues influence enzyme expression [13]. Especially light was shown to considerably impact cellulase gene expression in *T. reesei* and the gene set specific for inducing conditions is different in light and darkness [12, 14]. Light response in *T. reesei* is predominantly mediated by the photoreceptor complex consisting of BLR1 and BLR2 (blue light regulator 1 and 2) as well as by the photoreceptor ENV1 [15]. While BLR1 and BLR2 are GATA-type transcription factors, ENV1 is assumed to act on this complex by protein–protein interaction adjusting its function. All three components impact cellulase gene expression as well as transcript levels of numerous CAZyme encoding genes in *T. reesei* [16, 17]. The functions of ENV1 in *T. reesei* further extend to sexual and asexual development, growth and stress response [14].

Cyclic AMP (cAMP) is a central second messenger in the cellular function of all organisms and highly conserved across the kingdoms of life. The cAMP pathway crucially regulates morphology and nutrient sensing in fungi [18, 19].

The genome of *T. reesei* harbors an adenylate cyclase gene, an adenylate cyclase associated gene, two genes encoding catalytic subunits of protein kinase A (*pkac1* and *pkac2*), one regulatory subunit encoding gene and two phosphodiesterase genes [2]. Protein kinase A is the main target of cAMP signaling. PKAc1 was found to be the major catalytic subunit of protein kinase A in *N. crassa* [20] and plays an important role in circadian rhythmicity as well as stabilization of the photoreceptor complex WCC and the protein FREQUENCY (FRQ1) [21–23]. For *Aspergillus nidulans* a function of PkaA was found in biomass formation in complete media and on cellulose, endocellulase activity, expression of xylanases and cellulases [24]. Additionally, CreA is differentially phosphorylated depending on the presence of PkaA, resulting in altered characteristics for import in the nucleus [25]. A positive role of the cAMP pathway as well as the heterotrimeric G-protein pathway on cellulase gene expression and secondary metabolism was further shown in *Chaetomium globosum* [26, 27]. Besides functions in development, a role of the cAMP pathway in secondary metabolism was shown for *Aspergillus fumigatus*, *Pestalotiopsis microspora* and *Fusarium graminearum* [28–30].

**Fig. 1:**
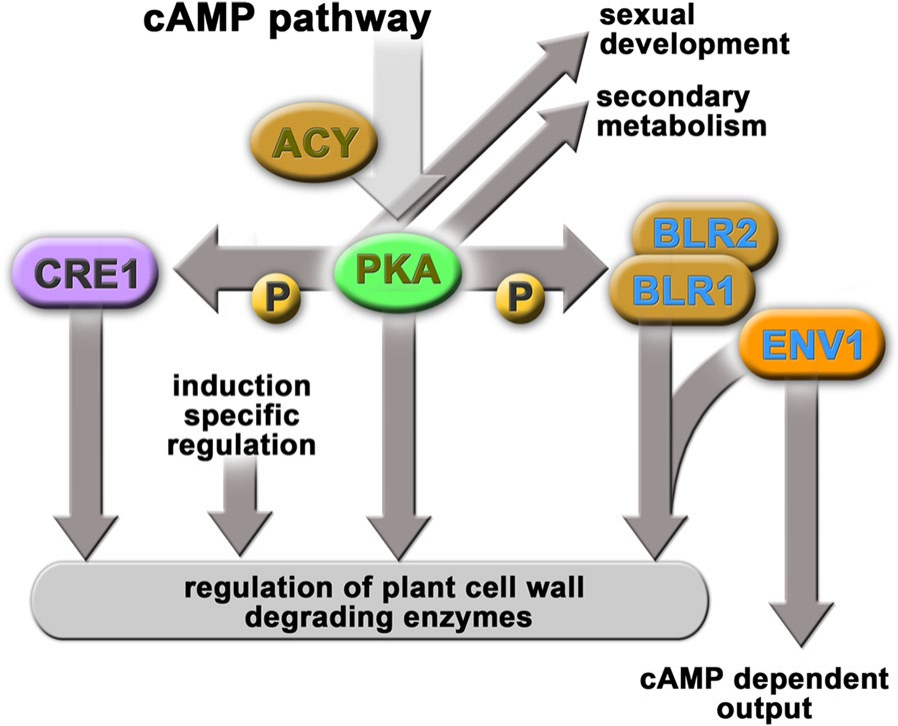
Model for the regulatory function of PKA in the cAMP pathway. Adenylate cyclase ACY impacts cAMP dependent function of PKA. The regulation of plant cell wall degrading enzymes by PKA is in parts mediated via phosphorylation of the carbon catabolite repressor CRE1 and the photoreceptor complex of BLR1 and BLR2. Function of the photoreceptor ENV1 on the BLR-complex overlaps with PKA regulation regarding enzyme production. The regulatory effect of ENV1 in part shows a cAMP level dependent output. PKA further positively impacts sexual development as well as production of secondary metabolites (for details see “Background”)

In *T. reesei* a broad physiological impact of the cAMP and the machinery adjusting its abundance is known [31–34]. The diverse functions of this pathway as shown in *T. reesei* and other fungi (Fig. 1) make protein kinase A an attractive target for investigation. The positive effect of cAMP on formation of endoglucanases in *T. reesei* has been described early in research on cellulase regulation [35] and confirmed with investigation of adenylate cyclase and protein kinase A [36]. Thereby, the cAMP level is modulated in response to the carbon source and is higher in the presence of the natural cellulase inducing compound sophorose than it is in the presence of cellulose, lactose, cellobiose or glucose, which corresponds to highest levels of cellulase gene expression on this carbon source [37]. The heterotrimeric G-protein pathway is one of the most important nutrient sensing pathways in fungi. In agreement with a carbon source dependent adjustment of cAMP levels, the G-protein alpha subunits GNA1 and GNA3 both influence intracellular cAMP content of *T. reesei* upon growth on cellulose [38, 39]. Interestingly, the impact of GNA1 and GNA3 as well as of *pkac1* and *acy1* is different in light and darkness under inducing conditions [36, 38, 39].

The light dependent functions of both the G-protein alpha subunits as well as of *acy1* and *pkac1* in *T. reesei* indicated a connection of the cAMP pathway to the light response pathway. Indeed, in a mutant lacking the photoreceptor ENV1, cAMP levels are strongly decreased. This phenotype is prevalent, even if GNA1 or GNA3 are constitutively activated in such a strain [40]. Additionally, genes regulated by ENV1 and ACY1, and hence likely reflecting the cAMP dependent output of the ENV1 regulon, show considerable overlap in light upon growth on cellulose [41]. Analysis of transcriptomes from different carbon sources suggests that this cAMP dependent regulatory output of ENV1 may be involved in substrate- or surface sensing [12]. The characteristic phenotype of strains lacking ENV1 (growth defect in light, altered sporulation [42, 43]) is reminiscent of both the perturbed growth of *acy1* and *pkac1* deletion strains and is hence likely to be cAMP related as well.

In this study we investigated the alterations in the transcriptome in dependence of the catalytic subunit 1 of protein kinase A (*pkac1*). Evaluation of the regulatory targets of PKAc1 showed functions in enzyme expression in light and darkness as well as modulation of secreted secondary metabolites upon growth on cellulose. Comparison of the PKAc1 regulome with those of known targets of PKA revealed considerable overlapping regulation, implicating the CAMK kinase GIN4 as potential target. Importantly, we also optimized a method for fungal secondary metabolite production by high performance thin layer chromatography (HPTLC) with a focus on large scale screening and metabolite quantification, using trichodimerol as representative compound.

## Results

### Transcriptome analysis of ∆*pkac1*

Previous studies showed that the cAMP pathway, including *pkac1*, is involved in regulation of cellulase gene expression in *T. reesei* upon growth on the cellulase inducing carbon source lactose [36]. Based on DNA–protein interaction studies and gene regulation analysis of the cellulase regulator *xyr1*, which is co-regulated with the cellobiohydrolase gene *cbh1* under these conditions, it was concluded, that PKAc1 acts on a transcriptional regulator impacting XYR1, rather than on XYR1 itself [36].

We investigated the transcriptome of ∆*pkac1* in comparison the the wildtype QM9414 upon growth on cellulose, which is more closely related to the natural substrate of *T. reesei* (Additional file 1). Due to the previously reported effect of light on the regulatory output of PKAc1 [36] and the functions of its homologues in *N. crassa* in regulation of the light response machinery as well as circadian rhythmicity [21, 22], we performed our experiments under controlled light conditions in constant light and constant darkness.

In order to validate the results of the transcriptome data we checked whether transcript levels of the wild-type correspond to known regulation patterns from previous studies [44]. The strongly light regulated genes *env1* and *hpp1* as well as TR_68924 and TR_123955 show the characteristic decrease in transcript levels in constant light compared to darkness. TR_123865 and TR_44278 show the characteristic increase in transcript abundance in light in the wild-type in our dataset. Hence we consider our transcriptome data valid and reliable.

### PKAc1 impacts metabolism and transport functions

**Fig. 2:**
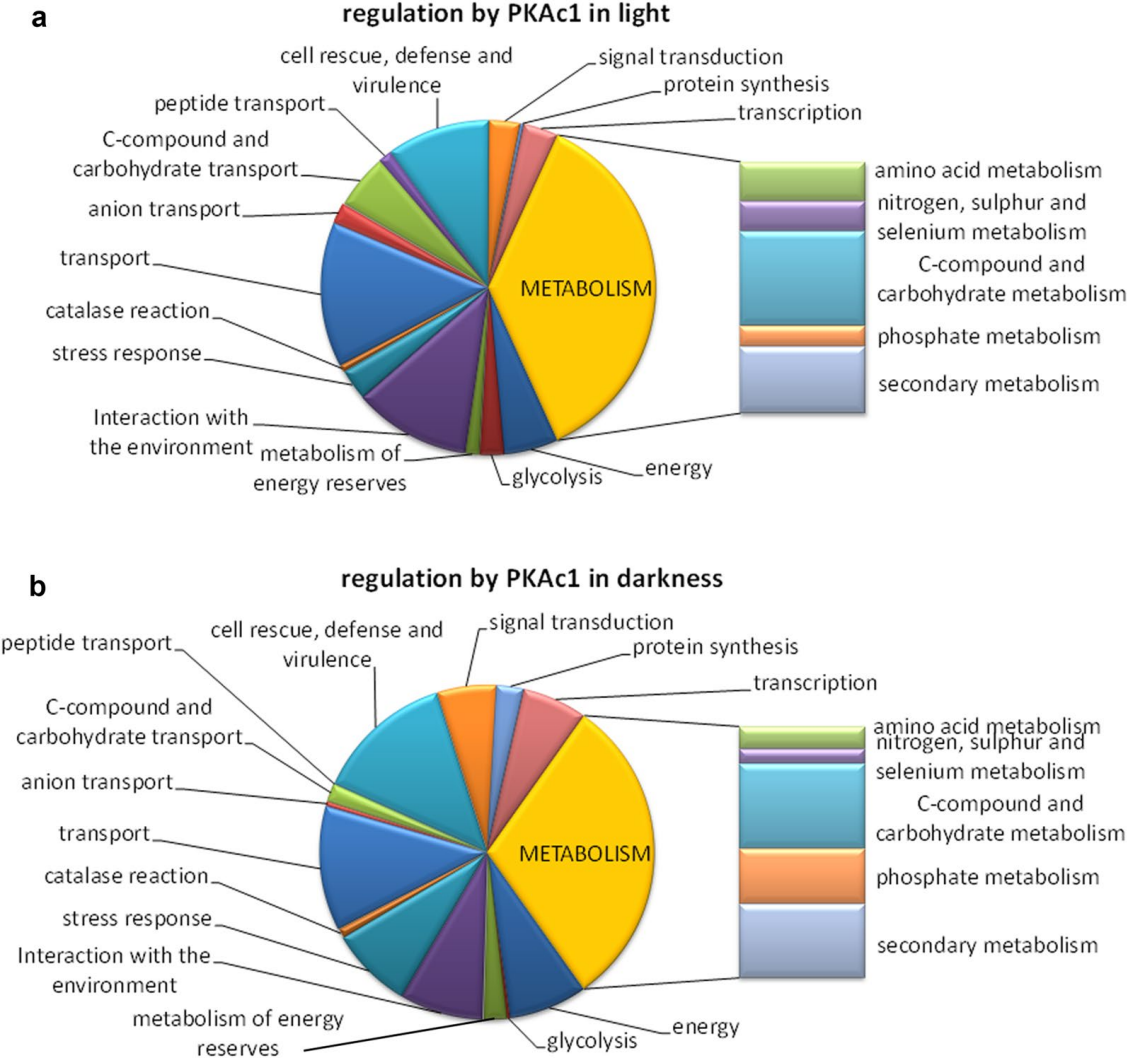
Functional category analysis of genes regulated by PKAc1 in **a** light or **b** darkness upon growth on cellulose in liquid culture. Selected, particularly relevant categories are shown

The regulatory targets of PKAc1 (indirect, as PKAc1 is not a transcription factor; Additional file 1) exert metabolic functions and comprise genes involved in signaling, transport and defense (Fig. 2a, b; Additional file 2). The genes regulated by PKAc1 in light are enriched in functions of transport (p-value 1.30E−04), particularly C-compound and carbohydrate transport (p-value 2.16E−05), nitrogen, sulphur and selenium metabolism (p-value 3.06E−4), C-compound and carbohydrate metabolism (p-value 9.7E−04) as well as in glycolysis and gluconeogenesis (p-value 2.68E−03). In darkness, the gene set regulated by PKAc is particularly enriched in glycogen catabolism (p-value 3.8E−04). Other metabolic functions as well as functions in energy supply are enriched to a lower extent (p-value < 5E−02).

### Genes regulated by PKAc1 in darkness

Constant darkness represents a condition closely related to the conditions of industrial fermentation and is hence most interesting for studying *T. reesei*. We found 170 genes to be more than twofold regulated (p-value threshold 0.01) in constant darkness compared to wildtype (Additional file 1). Among the 128 genes downregulated in darkness, we found 6 CAZyme encoding genes including a predicted trehalase as well as eight transcription factor genes, of which TR_122523 was previously shown to positively influence plant cell wall degradation [9]. TR_123881 (SxlR), which is more than threefold downregulated in ∆*pkac1*, represses xylanase, but not cellulase activity [45].

Interestingly, under these conditions the recently described SOR cluster, which is responsible for biosynthesis of dihydrotrichotetronine on cellulose [46] and impacts sorbicilline formation upon growth on glucose [47], is downregulated as well. The consistently positive effect of PKAc1 on the polyketide synthases (pks) *sor1* and *sor2*, the predicted flavoprotein monooxygenase gene *sor5*, the transporter *sor4* as well as the transcription factor *ypr2* (*sor3*) supports a regulation of this cluster via nutrient dependent phosphorylation. The second transcription factor gene in this cluster, *ypr1*, is not regulated by PKAc1 on cellulose, but positively regulated by the adenylate cyclase (ACY1) upon growth on cellulose in darkness [41]. For *ypr2*, the opposite is the case, it is not regulated by ACY1 [41]. Previously it was shown that the transcription factors YPR1 and YPR2 exert their functions in a different way on glucose and cellulose [46, 47]. Accordingly, carbon source dependent regulation of *ypr1* and *ypr2* is different between inducing and repressing carbon sources [12].

In darkness, only three genomic clusters of regulation were detected (Additional file 1), one of them being the SOR cluster on chromosome 5 of the recent genome sequence of QM6a [48].

The 43 genes upregulated in ∆*pkac1* in darkness included the small, cellulose specific gene *ooc1* [49], the polyketide synthase *pks4g*, which was shown to be responsible for the green spore coloration in *T. reesei* [50]. Additionally, transcript levels the gene represented by the model TR_64125, encoding a protein kinase were increased. Moreover, this gene set comprises the protein phosphatase gene *pp1* (TR_120722), which is related to *N. crassa* histidine phosphatase *pph*-*3* as well as two putative multicopper oxidases (TR_102820 and TR_124079) (Additional file 1). Of the two transcription factor genes upregulated in darkness in ∆*pkac1*, TR_112524 was screened for a function in plant cell wall degradation, which was however not detected [9].

### Genes regulated by PKAc1 in light

Upon growth in light with cellulose as carbon source, 225 genes were regulated compared to wild-type, 126 showed decreased transcript levels and 99 increased transcript levels in ∆*pkac1* (Additional file 1). Among the 126 genes downregulated in light, we found 9 CAZyme encoding genes including *gph1* encoding a glycogen phosphorylase regulated by Hog1 under stress conditions in *Candida albicans* [51] and the light dependent induction of which is positively regulated by ENV1. Additionally, this gene set comprises the gene encoding the mannitol dehydrogenase *lxr1* [52, 53] which resides in a gene cluster regulated by the photoreceptor ENV1 [17]. We also found downregulation of 5 genes associated to secondary metabolism, of them 4 cytochrome P450 encoding genes and 8 transcription factor genes of unknown function. The transcription factor encoding TR_54703 was tested for a positive function on plant cell wall degradation, but no alteration in enzyme expression was found [9].

Of the 6 transporter encoding genes regulated by PKAc1 in light, there is also *ste6*, encoding the putative pheromone transporter, which is downregulated in ∆*pkac1.* This is in agreement with the previously reported positive effect of the cAMP pathway on sexual development [36].

For 99 genes we detected upregulation in ∆*pkac1* in light. This geneset comprised 6 CAZyme including *cip2*, *cel3d* and *egl5*/*cel45a*, 9 genes involved in sulphur metabolism, among them 5 taurine dioxygenases and 3 methionine permeases. Moreover, we found 2 transcription factor encoding genes including *vib1* and TR_3449 as well as 3 transporters including MFS hexose transporter CLP1, which transports cellodextrins in *N. crassa* [54].

Interestingly, of the genes regulated by PKAc in light, *cip2* and the glycoside hydrolase family 30 xylanase TR_69276, which is assumed to have both endoxylanase and endoglucanase functionality [55] encode proteins that are listed among the three most important limiting proteins for hydrolysis of pretreated corn stover (PCS), a typical substrate for industrial enzyme production [55].

### Light independent targets of PKAc1

**Fig. 3:**
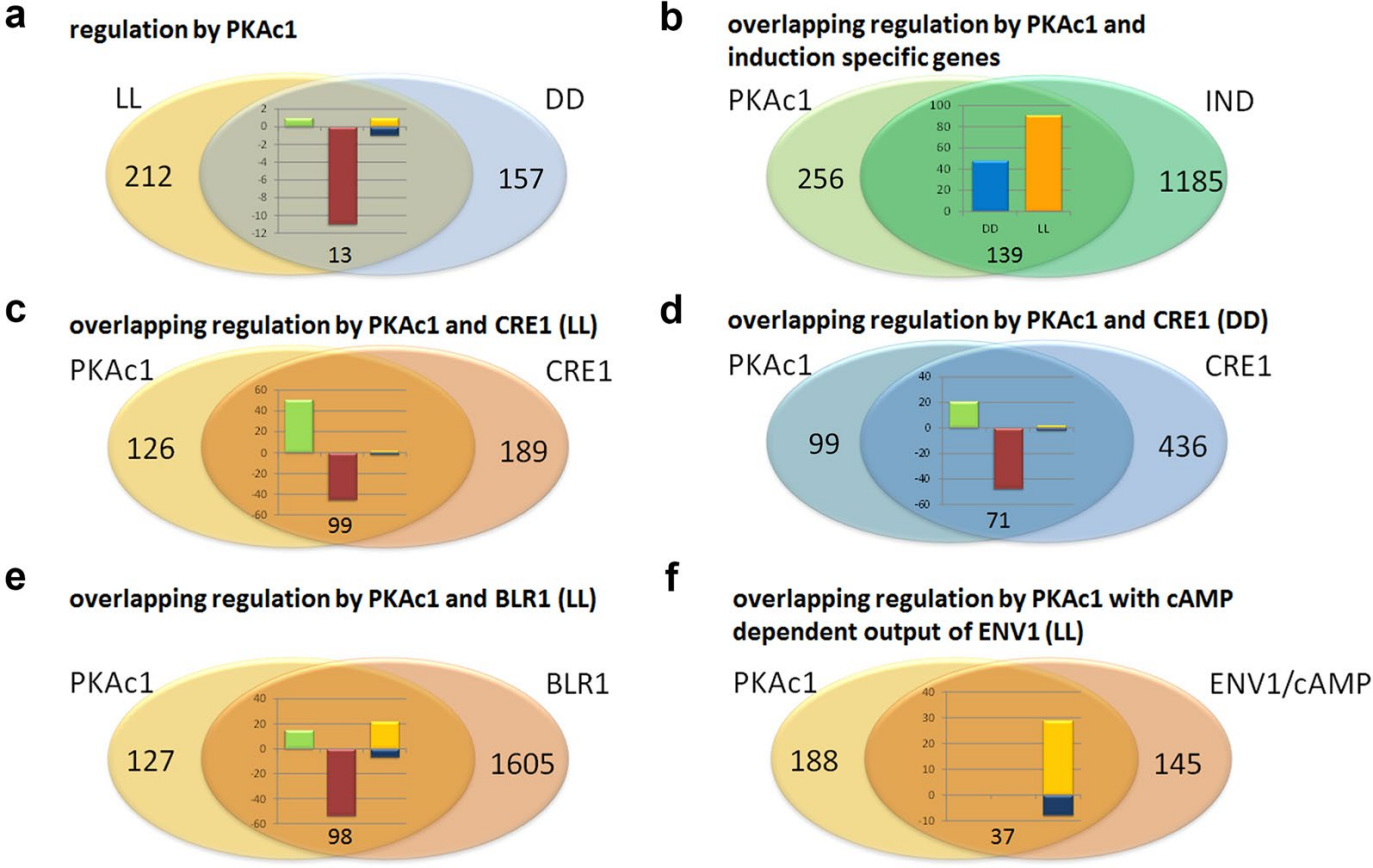
Genes regulated by PKAc1 and their correlation with patterns in other strains. **a** Overlap between genes regulated by PKAc1 in light (LL) and darkness (DD) is shown. **b** Overlap between induction specific genes [12] and genes regulated by PKAc1 is shown. Regulation in darkness (DD) is shown in blue, regulation in light is shown in yellow. **c** Overlap between genes regulated by PKAc1 and CRE1 [46] in light. **d** Overlap between genes regulated by PKAc1 and CRE1 [46] in darkness. **e** Overlapping regulation between PKAc1 and BLR1 [17] in light. **f** Overlapping regulation between the gene set reflecting the cAMP dependent output of the photoreceptor ENV1 [41] and regulation by PKAc1 in light. Genes consistently upregulated in the compared gene sets **a**, **c**–**f** are shown in green, those downregulated are shown in red. If a contrasting regulation was observed, the situation in ∆*pkac1* is shown in the figure (third column) with upregulation in yellow and downregulation in blue

Of the 382 genes regulated by PKAc1 upon growth on cellulose, only 13 genes showed regulation in both light and darkness, suggesting a considerable light dependent relevance for PKAc1 (Fig. 3a). The gene encoding the regulatory subunit of protein kinase A, *pkar1*, the CAZyme encoding gene TR_120198, the catalase gene *cat8* and the glycogen phosphorylase gene *gph1* are downregulated by PKAc1 in light and darkness. Of the two transcription factor genes regulated light-independently by PKAc1 (TR_105520 and TR_122523), TR_122523 influences cellulase regulation in *T. reesei* [9]. Contrasting regulation in light and darkness was found for TR_81122 (upregulated in light and downregulated in darkness) and TR_109378 is several fold upregulated in light and darkness in ∆*pkac1.*

### Induction specific genes regulated by PKAc1

Protein kinase A impacts diverse important physiological pathways in fungi (Fig. 1), often by phosphorylation of important transcription factors including photoreceptors [21] and the carbon catabolite repressor CRE1 [25]. Due to the relevance of PKAc1 in cellulase regulation [24, 36], a more general impact on induction specific genes is also expected. Therefore, we evaluated available transcriptomes from growth of *T. reesei* on cellulose in light and darkness for overlapping or contrasting regulation indicating functions in the same pathway.

Induction of plant cell wall degrading enzymes and more specifically of cellulases happens in response to distinct carbon sources, mainly representing plant cell wall components [56]. We were therefore interested if the core set of induction specific genes (regulation on the inducing carbon sources cellulose, lactose and sophorose versus glucose and glycerol, in the presence of which no cellulases are induced; [12]) in light and darkness is subject to regulation by PKAc1.

Of the 170 PKAc targets (indirect) in darkness, 48 overlapped with genes regulated in an induction specific manner [12], most of which were regulated in the opposite direction in ∆*pkac1* (positive vs. negative regulation; contrasting) (Additional file 3) (Fig. 3b). These overlapping genes were enriched in the function of catalase reaction (p-value 8.45e−04), but comprised also genes associated with stress response, metabolism including C-compound and carbohydrate metabolism and transport functions.

Among the 225 genes regulated by PKAc1 in light, 91 genes overlapped with the previously reported genes specifically regulated under inducing conditions [12] (Fig. 2c). As seen in darkness, in many cases the regulation in ∆*pkac1* was contrasting that under inducing conditions (Additional file 3). The function of catalase reaction was also enriched among the genes regulated by PKAc1 in light (p-value 3.12E−03) as were those involved in secondary metabolism (p-value 9.16E−03). Additionally, 37 genes associated with metabolism, particularly with C-compound and carbohydrate metabolism, amino acid metabolism, detoxification and transport functions were detected in this gene set.

The overlap of induction specific genes and those regulated by PKAc1 is limited to only six genes between light and darkness. Consequently, regulation of induction specific genes by PKAc is light dependent with distinct target gene sets. The contrasting regulation both in light and in darkness suggests that particularly induction specific up-regulation under the respective conditions is dependent on the function of PKAc1.

### Overlapping targets with CRE1

The carbon catabolite repressor CRE1 shows the potential for cAMP dependent phosphorylation in its protein sequence. Moreover, an influence on phosphorylation of CRE1 was recently shown for PKA in *A. nidulans* [25]. Therefore we screened the respective dataset for gene regulation by CRE1 in constant light and constant darkness upon growth on cellulose [46] for overlapping regulation with PKAc1 (Additional file 4).

Of the 225 genes regulated by PKAc in light, 99 were also regulated by CRE1 in light (Fig. 3c). Among these 99 genes, 46 were downregulated in ∆*pkac1* and in ∆*cre1* in light. Fifty-one genes were upregulated in ∆*pkac1* and in ∆*cre1* in light. They comprise *cip1* and *cel3d*, several putative permeases and genes involved in sulphur metabolism. However, two genes were upregulated in ∆*pkac1* and downregulated in ∆*cre1* in light.

In darkness, 48 genes were downregulated in ∆*pkac1* and ∆*cre1* (Fig. 3d), among them four CAZyme encoding genes, the catalase gene *cat2* and the superoxidedismutase gene *sod1*. Twenty-one genes were upregulated in ∆*pkac1* and ∆*cre1*, which comprise *ooc1* and *pks4* as well as the putative multicopperoxidase gene TR_124079. Only two genes showed contrasting regulation including the regulator of G-protein signaling (RGS) gene *rgs2* (TR_72259). These overlapping regulation patterns (Fig. 3c, d) support the hypothesis of a positive effect of phosphorylation by protein kinase A in the function of CRE1.

### Overlapping targets with photoreceptors

In *N. crassa*, a function for protein kinase A (PKA) in phosphorylation dependent regulation of the photoreceptor complex (white collar complex; WCC) activity was shown and PKA was found to serve as a priming kinase for the casein kinase dependent phosphorylation of the WCC components [57]. The *T. reesei* homologues of the WC photoreceptor complex, BLR1 and BLR2 [16], comprise putative cAMP dependent phosphorylation sites (data not shown). Hence, deletion of *pkac1* may in part cause similar gene regulation as lack of functionality or deletion of *blr1* or *blr2*.

Comparison of gene regulation by light in ∆*pkac1* with that in ∆*blr1* and ∆*blr2* [17] showed 98 of the 225 genes regulated by PKAc1 in light to be regulated by BLR1 in light as well (Fig. 3e) (Additional file 4). They include 15 CAZyme genes, 10 genes involved in sulfur metabolism, 10 transcription factor encoding genes including *vib1*, which was recently shown to regulate cellulase gene expression [58] and 9 genes encoding transporters.

Most of the genes overlapping between BLR1 targets and PKAc1 targets in light were consistently regulated in both mutants, but in some cases also contrasting regulation was observed (Fig. 3e). Of the 76 genes down regulated in ∆*blr1*, 22 were up-regulated in ∆*pkac1*. Interestingly, these genes comprise four CAZyme encoding genes including *cip2*, *cel3d* and *egl5* as well as the hexose transporter gene *clp1*, which is located next to *cel3d* in the genome.

Among the 22 genes up regulated in ∆*blr1*, 7 were down-regulated in ∆*pkac1*. They include three transcription factors (TR_120975, TR_122523 and TR_105220). TR_122523 was previously shown to positively influence cellulase gene expression and to be co-expressed with many hemicellulase genes [9].

Many of the genes with regulation in ∆*pkac1* and ∆*blr1* in light are also regulated in ∆*blr2* (Additional file 4). Ninety of the 225 genes were regulated by both. Again 39 of the genes down regulated in ∆*blr2* showed contrasting regulation in ∆*pkac1*, with 7 genes involved in sulphur metabolism. Up-regulated genes in ∆*blr2*, which are down-regulated in ∆*pkac1* comprise the same transcription factor genes showing contrasting regulation with ∆*blr1*. Protein kinase A was shown to inhibit the activity of the photoreceptor complex by acting as a priming kinase in *N. crassa* [21]. Hence deletion of protein kinase A would lead to increased photoreceptor activity. Our findings show a complex picture in this respect. Positive regulation of transcription factors by the photoreceptor complex BLR1–BLR2 (up-regulation in the mutants) occurs in the wildtype. Under these conditions, inhibition of the photoreceptor complex (to some extent) by PKA is operative. Deletion of *pkac1* should alleviate the negative effect on BLR1 and BLR2 activity and the regulation by the photoreceptor complex should remain or even increase. This is the case for two transcription factor genes (TR_71823 and TR_105980). However, we also saw the opposite effect for three transcription factors (TR_120975, TR_105220 and TR_122523), potentially reflecting an indirect effect of the presence of *pkac1* on a downstream component of the regulatory cascade of BLR1 and BLR2.

Functional category analysis of genes with altered transcript levels in ∆*pkac1*, ∆*blr1* and ∆*blr2* in light, indicating regulation by an effect of PKAc1 on activity of the photoreceptor complex showed enrichment (p-value threshold < 5e−02) in nitrogen, sulfur and selenium metabolism (p-value 8.48e−3), C-compound and carbohydrate transport (p-value 8.19e−04) and detoxification involving cytochrome P450 (p-value 3.0e−03) (Additional file 2).

### The cAMP dependent output of ENV1

Deletion of *env1* causes strongly decreased cAMP levels and a severe growth defect in light [40, 43]. Comparison of the transcriptional targets of ENV1 with those of the adenylate cyclase ACY1, revealed an overlap of 31 genes up-regulated in light and 114 genes down regulated in light, which represent the cAMP dependent regulatory targets of the photoreceptor ENV1. No genes with contrasting regulation in the two mutants were found [41]. In part these genes also correlate with genes implicated in surface sensing in *T. reesei* [12].

Of the respective gene set down regulated in light in ∆*env1* and ∆*acy1*, 29 of 114 genes were also regulated by PKAc1, but surprisingly all of them were upregulated in ∆*pkac1* (Fig. 3f) (Additional file 4). They include the CAZyme encoding genes *cip1* and *egl5*, four genes involved in sulphur metabolism as well as the transcription factor *vib1*. Also the 8 genes which overlap with the 31 genes up-regulated in ∆*acy1* and ∆*env1* in light, show contrasting regulation in ∆*pkac1*. We conclude that part of the cAMP dependent regulatory output of ENV1 in light is mediated by PKAc1, including the important transcription factor gene *vib1*. The overlapping regulation also with the photoreceptors BLR1 and BLR2 emphasizes the light dependent function of VIB1 in light dependent substrate sensing and cellulase regulation, which remains to be studied in detail.

### GIN4 moderately influences trichodimerol levels

Due to the positive influence of PKAc1 on SOR cluster genes, we checked regulated genes overlapping with the CRE1 regulon to select a potential regulator of sorbicillin production. TR_64125, encoding a homologue of the CAMK kinase GIN4 is up-regulated in ∆*pkac1* (2.8fold) and in ∆*cre1* (20-fold) in darkness. Additionally, transcript abundance of this gene is more than twofold significantly decreased in a strain lacking the SOR-cluster transcription factor YPR2 under the same conditions [59].

**Fig. 4:**
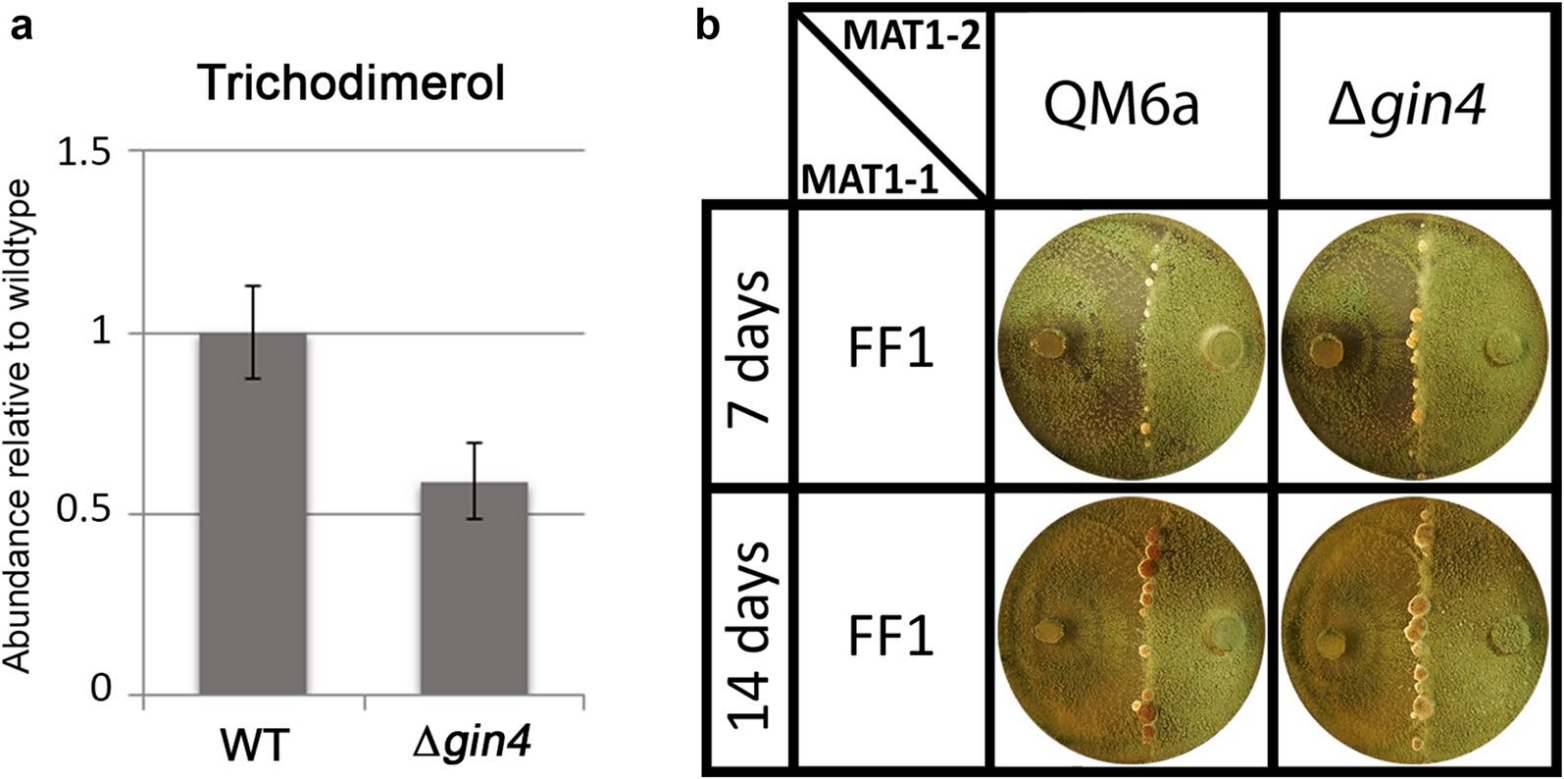
Influence of *gin4* on trichodimerol production and fruiting body development. **a** Trichodimerol production in cellulose liquid culture in total darkness of ∆*gin4* relative to wildtype (QM6a∆*mus53*) (p-value = 0.075). **b** Sexual development of ∆*gin4* and QM6a with FF1 after 7 and 14 days

Analysis of a deletion strain of *T. reesei* GIN4 for production of trichodimerol representing the compounds associated with the SOR cluster showed only a moderate alteration of trichodimerol levels (Fig. 4a). Lack of *A. nidulans* Gin4 in the genome causes earlier sexual development [60]. Hence we analyzed mating behaviour of ∆*gin4*, which revealed no significant difference to the wildtype in timing for fruiting body formation or morphology (Fig. 4b).

### Establishing a method for large scale screening of fungal secondary metabolite patterns

We found that PKAc1 is involved in regulation of the SOR cluster at the transcript level. Previously, genes of this cluster were shown to be among the most strongly transcribed genes under conditions of sexual development, considerably exceeding the transcript levels on cellulose [61]. Therefore, we aimed to cross-check the impact of PKAc1 on production of sorbicillin compounds under mating conditions. Deletion of *pkac1* causes delayed fruiting body formation in *T. reesei* [36] and *N. crassa* [62], indicating a positive effect of the cAMP pathway on sexual development, although it is not essential for mating [36].

First, we aimed to isolate a representative sorbicillin compound as a reference in our analyses. Then we optimized secondary metabolite extraction and the previously tested method of high performance thin layer chromatography (HPTLC) to establish a method for large scale screening of samples from diverse fungal strains and environmental conditions.

HPTLC allows for analysis of patterns of secreted metabolites of multiple samples (tens to even hundreds within reasonable time), which can be directly visually compared without employing complex and costly metabolomics tools like mass spectrometry. Reliable identification and quantification of individual compounds based on authentic standards is possible [63, 64]. The provided workflow also allows extraction of liquid samples from culture filtrates—ideally related to the produced biomass. Consequently, we consider HPTLC as the optimal system for screening of mutant libraries and the evaluation of diverse conditions for selection of samples for in depth analysis by mass spectrometry.

### PKAc1 impacts trichodimerol production

Crossing plates containing the wild-type strains FF1 and FF2 [65], both sexually fertile, were extracted with a focus on potential sorbicillinoids. After chromatographic purification, NMR analysis confirmed the identity of the sorbicillin derivative trichodimerol, production of which is associated with the SOR cluster [46]. Trichodimerol was produced under conditions of sexual development and therefore reflects chemical communication events between the two (potential) mating partners. Hence, trichodimerol can be used as a reference in high performance thin layer chromatography (HPTLC) analyses for secondary metabolite production during sexual development.

**Fig. 5:**
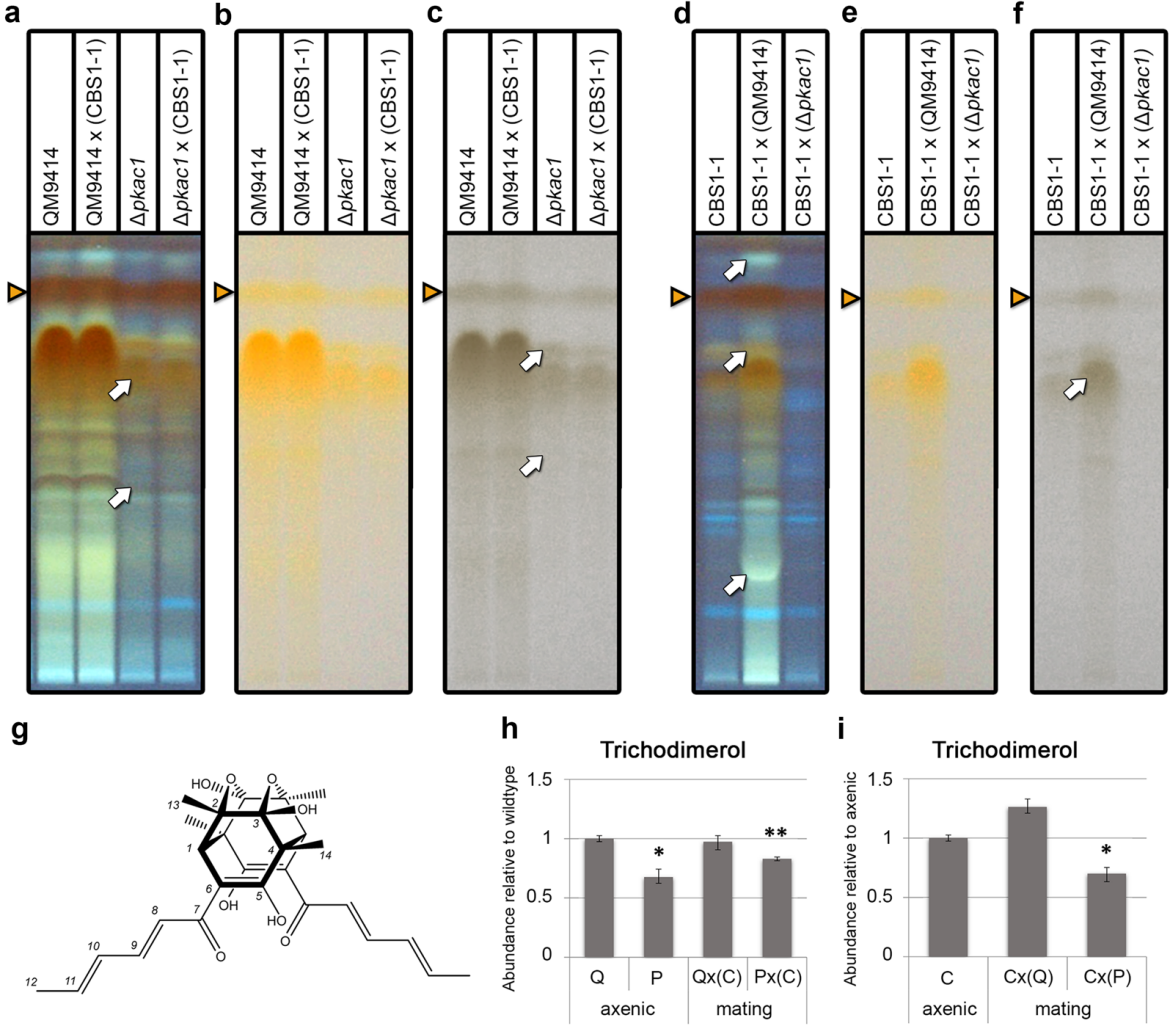
Detection and quantification of trichodimerol. **a**–**f** High performance thin layer chromatography (HPTLC) analysis of ∆*pkac1.* Triangles show trichodimerol. Arrows highlight major differences between samples. Secondary metabolite patterns of ∆*pkac1* and wildtype QM9414 under asexual and crossing conditions (**a**–**c**) and reaction of CBS1-1 after 14 days (**d**–**f**) on 2% MEX at 22 °C, LD. Visualization: **a**, **d** fluorescence at 366 nm, **b**, **e** visible light, **c**, **f** visible light with low saturation for better illustration. Analyses were done in three biological replicates with tree pooled plates per replicate. Replicates for HPTLC analysis were consistent and are provided in Additional file 5. **g** Trichodimerol. HR ESI–MS *m/z* 497.2164 [M+H]^+^ (calcd for C_28_H_33_O_8_, 497.2175), *m/z* 519.1994 [M+Na]^+^ (calcd for C_28_H_32_O_8_Na, 519.1995); ^1^H NMR (600 MHz, CD_3_OD): δ_H_ = 7.29 (1H, dd, *J* = 14.9 Hz, *J* = 10.9 Hz, H-9), 6.39 (1H, dd, *J* = 15.0 Hz, *J* = 10.9 Hz, H-10), 6.35 (1H, d, *J* = 14.9 Hz, H-8), 6.24 (1H, dq, *J* = 15.0 Hz, *J* = 7.0 Hz, H-11), 3.11 (1H, s, H-1), 1.92 (3H, d, *J* = 7.0 Hz, H-12), 1.40 (3H, s, H-14), 1.38 (3H, s, H-13); ^13^C NMR (150 MHz, CD_3_OD): δ_C_ = 201.3 (s, C-5), 175.8 (s, C-7), 144.1 (d, C-9), 140.8 (d, C-11), 132.7 (d, C-10), 120.2 (d, C-8), 105.7 (s, C-3), 104.6 (s, C-6), 80.3 (s, C-2), 60.9 (s, C-4), 58.6 (d, C-1), 21.7 (q, C-13), 19.8 (q, C-14), 18.7 (q, C-12). Numbering of protons and carbons is shown. All data in agreement with those reported earlier for this compound [79]. **h** Quantification of trichodimerol in axenic growth in the parental strain QM9414 (Q) and in ∆*pkac1* (P) compared to interaction with the fully fertile strain CBS999.97 MAT1-1 (C) under conditions favoring sexual development (corresponds to HPTLC data on panel D). **i** Quantification of trichodimerol in axenic growth in CBS999.97 MAT1-1 compared to interaction with QM9414 and ∆*pkac1* under conditions favoring sexual development (corresponds to HPTLC data on panel **c**). Error bars reflect standard deviations, *p-value < 0.05 and **p-value < 0.01

To investigate secondary metabolite production upon sexual development we grew ∆*pkac1* alone and in the presence of a mating partner. Additionally, we tested the response of a wild-type strain to the encounter of ∆*pkac1*. Wildtype combinations were used as controls (Fig. 5a–f). This analysis showed that already in the absence of a mating partner, secondary metabolite production of ∆*pkac1* was decreased compared to wildtype (Fig. 5a–c, arrows) including an impact on potential sorbicillin derivatives. For the dimeric sorbicillin derivative trichodimerol (Fig. 5g) as representative of sorbinillinoids (Fig. 5a–f, triangles) a decrease by 32.4% (± 6.7%, p-value 0.012) was detected (Fig. 5h). The reaction of both wildtype and ∆*pkac1* to the presence of CBS999.97 MAT1-1 as mating partner was only subtle (Fig. 5a–c, arrows). In contrast, the reaction of the fully fertile strain CBS999.97 MAT1-2 to the parental strain of ∆*pkac1*, QM9414 was very clear compared to axenic growth of this strain (Fig. 5d–f, arrows). Also potential sorbicillin compounds were concerned (Fig. 5f) and trichodimerol showed a positive trend. Deletion of *pkac1* almost abolished this response and resulted in a lower abundance of trichodimerol compared to axenic growth by 30.1% (± 9.3%, p-value 0.028) (Fig. 5i). Consequently, the association of the SOR cluster and its products with chemical communication during sexual development is supported as well as a role of PKAc1 in this process.

## Discussion

The cAMP pathway represents one of the most important signaling checkpoints in living organisms, with protein kinase A being a central component of the signal transmission machinery. Due to its function in adjustment of the levels of the secondary messenger cAMP, this pathway was postulated to be a coincidence detector [66] or a means to integrate signals from different sources. The components of this pathway as well as cAMP itself were previously shown to impact cellulase regulation [67] in a concentration dependent manner [35]. Additionally, considerable light dependent effects in correlation with the cAMP pathway were observed including altered membrane potential, intracellular levels of ATP and cAMP and an increase of oxygen consumption [68]. cAMP dependent protein kinase A is an important output pathway of altered cAMP levels and represents one of the factors mediating the physiological effects of changing cAMP concentrations in the cell.

Besides an influence on regulation of several CAZyme encoding genes, our data showed that PKAc1 also impacts glycogen metabolism. Interestingly, several genes involved in metabolism of energy reserves like glycogen or trehalose are downregulated in ∆*pkac1*, for example the glycoside hydrolase family 31 encoding TR_82235 and the putative trehalase TR_123456. The glycosylphosphorylase gene *gph1*, (glycosyltransferase family 35; TR_120198), is down regulated as well in light and darkness in ∆*pkac1.* This gene is located between the G-protein alpha subunit gene *gna3* and the MAPkinase gene *tmk3* [38] and is regulated by light and ENV1 in *T. reesei* [69]. Moreover, growth on glycogen as carbon source is decreased in a mutant lacking ENV1, but not in wildtype in light [69]. The regulatory output of ENV1 is in part mediated by adjustment of cAMP levels [40, 41], particularly in light. Also gene regulation in ∆*pkac1* overlaps with the gene set representing the cAMP dependent output of ENV1. Intriguingly, all these genes show contrasting regulation in ∆*pkac1* compared to ∆*env1* and ∆*acy1* (Fig. 2g). Consequently, the cAMP dependent output of ENV1 involves the function of PKAc1 as well. Moreover, the previously reported involvement of the cAMP pathway in energy supply of the cell and adjustment of reserve carbohydrates is in agreement with the function of PKAc1, which positively influences genes involved in degradation of glycogen and trehalose.

Besides the connection of PKAc1 to the photoreceptor ENV1, also the considerable overlap of gene regulation by PKAc1 with the components of the photoreceptor complex BLRC, BLR1 and BLR2, strengthens the importance of PKA in dealing with altered light conditions in *T. reesei*.

One of the most interesting genes in this PKAc1 mediated, cAMP dependent output of ENV1 is the regulator gene *vib1*. In *N. crassa*, VIB1 links glucose signaling to carbon catabolite expression and is required for cellulase gene expression, due to its impact on the transcription factor CLR2 [70]. VIB1 impacts expression of genes involved in metabolism and energy and may hence mediate the effects we saw for PKAc1. Accordingly, VIB1 is a key regulator of cellulase gene expression in *T. reesei* [58]. However, *T. reesei* CLR2 does not have a striking function in cellulase regulation in *T. reesei* [9], indicating rewiring of the pathway.

Secondary metabolism in *T. reesei* is known to be modulated in light upon growth on cellulose [46]. We isolated trichodimerol as a representative of sorbicillins, which are enhanced under conditions of sexual development [61] and demonstrate its regulation by PKAc1. Together with the refined HPTLC method for quantification, we thereby provide a means to screen large numbers of samples for modifications in trichodimerol production, which can also serve as a representative for metabolites associated with the SOR cluster including sorbicillins in general. This method is valuable as a prescreening for in depth analysis of selected samples by mass spectrometry. In addition, the presented method is applicable for extraction of secondary metabolites from supernatants of liquid cultures in relation to the biomass produced by the organism.

The genes associated with secondary metabolism regulated by PKAc1 comprise the particularly interesting secondary metabolite cluster responsible for biosynthesis of sorbicillinoids (SOR cluster). *Ypr2*, encoding one of the transcription factors regulating the SOR cluster, is regulated by light [59], by PKAc1 in darkness and by BLR1 and BLR2 in light. Previously we found a positive feedback cycle for regulation of the SOR cluster in darkness, which may involve the function of YPR2 [46]. Since the genes of the SOR cluster are considerably upregulated under mating conditions [61] and the production of sorbicillins including trichodimerol and dihydrotrichotetronine on cellulose is low, we chose to evaluate the role of PKAc1 in secondary metabolism in combination with sexual development. Therefore we isolated trichodimerol as a representative for the SOR cluster.

Besides a decrease in trichodimerol production and biosynthesis of putative sorbicillinoids in general we also found that in ∆*pkac1* communication with a putative mating partner on the plate was altered. Interestingly, the response to a wildtype strain, that was elicited in the fully fertile CBS999.97 strain in a confrontation assay, was not observed with ∆*pkac1.* In addition to a certain defect in sexual development in ∆*pkac1* as shown previously [36], this signaling alteration represents a further function of PKAc1 in development.

## Materials and methods

### Strains and culture conditions

QM9414 (ATCC26921) and ∆*pkac1* [36] were used for transcriptome analysis. FF1 and FF2 [65] were used for analysis of development and CBS999.97 MAT1-1 [71] was used to study secondary metabolite production and chemical communication. For transcriptome analysis, strains were grown in 200 ml Mandels Andreotti minimal medium [72] with 1% (w/v) cellulose (Alfa Aesar, Karlsruhe, Germany) as carbon source in constant light (1800 lx) or constant darkness at 28 °C on a rotary shaker (200 rpm) for 72 h. Dark grown cultures were harvested under red safety light (darkroom light, Philips PF712E) to prevent any influence of a light pulse on transcript levels.

For HPTLC analysis, strains were cultivated on 2% malt extract agar medium at 22 °C, LD (12 h light, 12 h darkness) at 1700 lx until harvest after 14 days. Petri dishes were inoculated near the margin with agar slices of 0.5 × 4 cm from fully grown cultures to guarantee an even confrontation line.

### Transcriptome analysis

For transcriptome analysis, custom arrays were used with the gene expression service as provided by Roche-NimbleGen (Madison, USA). Therefore, total RNA was isolated and quality controlled as described previously [40, 44]. Data of two biological replicates are deposited at NCBI Gene Expression Omnibus (GEO accession number GSE131419). Bioinformatic analysis was done using the PARTEK Genomics Suite 6.6 (St. Louis, USA) applying a threshold of twofold regulation and a p-value of 0.01 (false discovery rate (FDR) corrected; ANOVA statistics). Functional category analysis was done using the FUNGIFUN2 online tool [73]. Genomic clustering was evaluated using the open source software REEF [74] and adjusted manually.

### Construction and analysis of ∆*gin4*

Deletion of *gin4* (TR_64125) was performed as described previously [75] using yeast mediated recombination for vector construction and protoplast transformation for homologous integration into QM6a∆*mus53* [76] and the hygromycin phosphotransferase cassette as selection marker. Successful deletion of *gin4* was confirmed by PCR with primers binding within the deleted region. DNA integrity appropriate for PCR amplification of the samples representing *gin4* was ensured using standard primers amplifying the *tef1* gene. Sexual development was investigated under standard conditions [71, 77]. ∆*gin4* was grown on minimal medium with cellulose as carbon source as described above in constant darkness. Trichodimerol production in culture filtrates was analyzed by mass spectrometry using internal standards as described previously [46, 78].

### High-performance thin-layer chromatographic (HPTLC) analysis and sample preparation

**Fig. 6:**
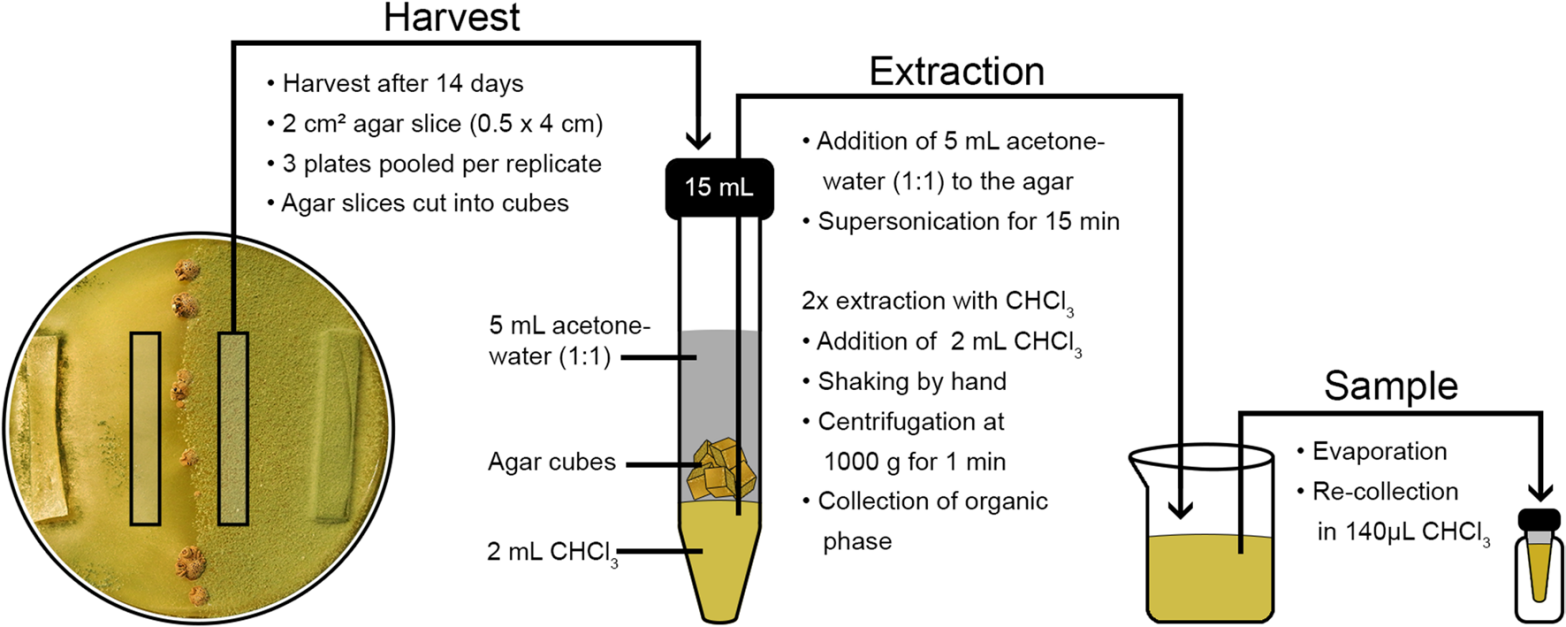
Workflow of sample preparation for high performance thin layer chromatography (HPTLC) analysis from fungal cultures grown on agar medium

Analysis was done according to Bazafkan et al. [65] with modifications. The workflow for preparation of analytical samples from fungal cultures grown on agar medium is shown in Fig. 6. All samples were measured in three biological replicates from three pooled plates each. Agar slices of 2 cm^2^ (0.5 × 4 cm) were collected near the confrontation zone in crossings and from the corresponding area in asexual cultures. Metabolites were extracted from collected agar slices in 15 ml centrifugation tubes by addition of 5 ml 50% acetone in water and supersonication for 15 min. Thereafter 2 ml chloroform (CHCl_3_) were added, the tube shaken by hand and centrifuged at 1.000*g* for 1 min for phase separation. Organic phase was collected in glass vials for evaporation. CHCl_3_ extraction was repeated two times. Dry extracts were re-collected in 140 µl CHCl_3_ and 5 µl applied to HPTLC analysis. Samples were spotted onto a normal phase silica gel plate (HPTLC silica gel 60 F254s, glass, 200 × 100 mm, Merck, Darmstadt, Germany, #1.1.5696.0001) with an automatic TLC sampler (ATS 4, CAMAG, Muttenz, Switzerland) with 4.5 mm band length and 5.5 mm track distance for 30 samples per plate. As mobile phase a mixture of water extracted CHCl_3_ and 1 mM trifluoroacetic acid in methanol 7:1 (v/v) was used. Developing was done in an automated developing chamber (ADC2, CAMAG, Muttenz, Switzerland) at a relative humidity of 11% and a migration distance of 70 mm. Metabolite patterns were analyzed at 254 nm, 366 nm and visible light with a TLC visualizer (CAMAG, Muttenz, Switzerland) before and after derivatization with p-anisaldehyde:sulfuric acid reagent. Scanning densitometry was done with a TLC scanner (Scanner 3, CAMAG, Muttenz, Switzerland) at various wavelengths (254, 290, 345, 366, 420, 470 and 520 nm) before derivatization. Results were recorded and evaluated using the software visionCATS 2.0 (CAMAG, Muttenz, Switzerland).

### Detection and quantification of trichodimerol using HPTLC

Trichodimerol was identified in secondary metabolite samples by comparing the Rf-value and UV/vis spectra (200–800 nm; Scanner 3, CAMAG, Muttenz, Switzerland) to those of the isolated reference compound. Trichodimerol was quantified relative to wildtype. A linear calibration based on peak height at 420 nm was obtained from combined wildtype replicates and two dilutions (1:3; n = 3). Significance was evaluated by t-test in RStudio (compare_means, ggpubr).

### Isolation of trichodimerol

Well grown FF1 and FF2 plates containing 3% (w/v) malt extract agar were extracted with an excess amount of a mixture of chloroform and acetone (1:1) and sonication for 15 min. The solvent mixture was filtered and further purified by two times liquid–liquid extraction with water. The solvents of the organic phase were evaporated and the crude extract stored at − 20 °C until processing.

Size-exclusion column chromatography of 126 mg crude extract over Sephadex LH-20^®^ [GE Healthcare; 750 mm column length, 12 mm diameter, coupled with C-640 UV detector (Büchi)] eluted isocratically with methanol yielded 16 mg of impure trichodimerol. Final purification of this fraction was accomplished by preparative thin layer chromatography (TLC) using silica gel 60 glass plates (PLC Silica gel 60 F_254_, 0.5 mm thickness; Merck, Darmstadt, Germany) developed in dichloromethane/methanol (98:2). This step afforded 7.2 mg trichodimerol.

### NMR spectroscopy

For NMR spectroscopic measurements trichodimerol was dissolved in CD_3_OD (~ 3.0 mg in 0.7 mL) and transferred into 5 mm high precision NMR sample tubes. All spectra were measured on a Bruker DRX-600 at 600.25 MHz (^1^H) or 150.94 MHz (^13^C) and performed using the Topspin 3.5 software (Bruker, Rheinstetten, Germany). Measurement temperature was 298 K ± 0.05 K. 1D spectra were recorded by acquisition of 32 k data points and after zero filling to 64 k data points and Fourier transformation spectra were performed with a range of 7200 Hz (^1^H) and 32,000 Hz (^13^C), respectively. To determine the 2D COSY, TOCSY, NOESY, HMQC, and HMBC spectra 128 experiments with 2048 data points each were recorded, zero filled and Fourier transformed to 2D spectra with a range of 6000 Hz (^1^H) and 24,000 Hz (HSQC) or 32,000 Hz (HMBC) (^13^C), respectively. Residual CD_2_HOD was used as internal standard for ^1^H NMR measurements (δH 3.34) and CD_3_OD for ^13^C NMR measurements (δC 49.0).

### Mass spectrometry

Mass spectra were measured on a high resolution time-of-flight (hr-TOF) mass spectrometer (maXis, Bruker Daltonics) by direct infusion electrospray ionization (ESI) in positive ionization mode (mass accuracy ± 5 ppm). TOF MS measurements have been performed within the selected mass range of *m/z* 100–2500. ESI was made by capillary voltage of 4 kV to maintain a (capillary) current between 30 and 50 nA. Nitrogen temperature was maintained at 180 °C using a flow rate of 4.0 l min^−1^ and the N_2_ nebulizer gas pressure at 0.3 bar.

## Supplementary information

**Additional file 1.** Target genes of PKAc1 during growth on cellulose in light and darkness.

**Additional file 2.** Functional category analysis of genes with altered transcript levels in Δ*pkac1* and the subset of genes with overlapping regulation to photoreceptor targets.

**Additional file 3.** Genes with altered transcript levels in Δ*pkac1* and regulation in an induction specific manner.

**Additional file 4.** Comparative analysis of genes with altered transcript levels in Δ*pkac1* and genes regulated by photoreceptors, CRE1 and by light upon growth on cellulose.

**Additional file 5: Figure S1.** Biological replicates of high performance thin layer chromatography (HPTLC) analysis of wildtype and *pkac1* mutants under asexual and sexual conditions.

## Data Availability

All data generated during this study are included in this published article and its additional files. GenBank Accession numbers for datasets analyzed for this study are given in Methods and described in the respective cited articles.
