# Supplementary material for: The role of PKAc1 in gene regulation and trichodimerol production in Trichoderma reesei
Source: Fungal Biol Biotechnol. 2019 Sep 10;6:12. doi: 10.1186/s40694-019-0075-8 (PMC6734591; doi:10.1186/s40694-019-0075-8)
Supplement: Supplementary file 5 — Additional file 5: Figure S1. Biological replicates of high performance thin layer chromatography (HPTLC) analysis of wildtype and pkac1 mutants under asexual and sexual conditions. [file 40694_2019_75_MOESM5_ESM.pdf]

The role of PKAc1 in gene regulation and trichodimerol production in *Trichoderma reesei*

Wolfgang Hinterdobler[1], André Schuster[2], Doris Tisch[2], Ezgi Özkan[1,3], Johann Schinnerl[4], Lothar Brecker[5], Stefan Böhmdorfer[3], and Monika Schmoll[1]*

SUPPLEMENTARY MATERIAL

Figure S1

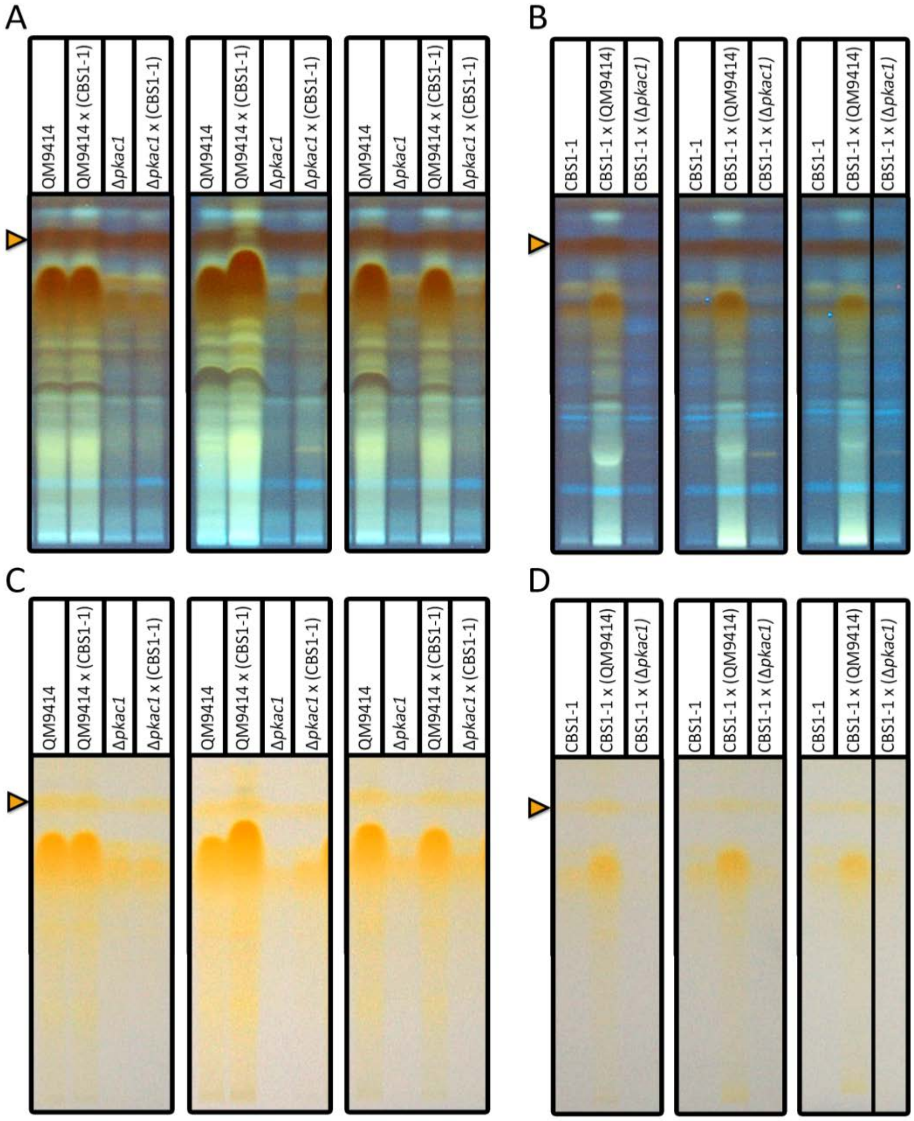

Figure S1. **Biological replicates of high performance thin layer chromatography (HPTLC) analyses**. Triangles show trichodimerol. Secondary metabolite patterns of Δ*pkac1* and wildtype QM9414 under asexual and crossing conditions (**A**, **C**) and reaction of CBS1-1 (**B**, **D**) after 14 days on 2 % MEX at 22 °C, LD. Visualization: (**A**, **B**) fluorescence at 366 nm, (**C**, **D**) visible light. Third replicate (right) was measured in a different sequence order for closer comparison between samples and is therefore shifted in (**A**, **C**) and one lane removed (indicated by black line) in (**B**, **D**). Samples from three petri dishes were pooled for each replicate.
